# Impact of immune cells on the risk of frozen shoulder: A 2-sample Mendelian randomization study

**DOI:** 10.1097/MD.0000000000040271

**Published:** 2024-11-01

**Authors:** Yinji Luo, Xinyu Wang, Bin Wang

**Affiliations:** a Department of Bone Surgery, The Second Affiliated Hospital, Guangzhou Medical University, Guangzhou, Guangdong Province, China; b Zhongshan School of Medicine, Sun Yat-sen University, Ministry of Education, Guangzhou, Guangdong Province, China.

**Keywords:** frozen shoulder, genome-wide association studies, immune cells, Mendelian randomization, SNPs

## Abstract

The pathogenesis of frozen shoulder (FS) remains unclear, and current research primarily focuses on immune responses. Increasing evidence suggests that immune cells play a significant role in FS development. However, the causal relationship between the two remains poorly understood. Therefore, we aimed to investigate this using Mendelian randomization (MR) analysis. Single nucleotide polymorphisms closely associated with 731 immune phenotypes were obtained from publicly available GWAS datasets as instrumental variables. FS was used as the outcome with a sample size of 451,099 cases. Causal effects were analyzed using the inverse variance-weighted method. We conducted sensitivity tests, including the intercept of the MR-Egger and MR-PRESSO analyses. The presence of heterogeneity was evaluated using Cochran *Q* test. We identified potential causal relationships in terms of increased risk for FS with 5 immune phenotypes: CD25++ CD45RA+ CD4 not regulatory T cell %CD4+ T cells (odds ratio [OR] = 1.0273, 95% confidence interval [CI]: 1.0093–1.0457, *P* = .0028), CD25++ CD45RA+ CD4 not regulatory T cell %T cell (OR = 1.0240, 95% CI: 1.0057–1.0427, *P* = .0098), CD127 on CD28+ CD4+ T cells (OR = 1.0398, 95% CI: 1.0121–1.0682, *P* = .0046), CD4 on human leukocyte antigen DR+ CD4+ T cells (OR = 1.0795, 95% CI: 1.0316–1.2195, *P* = .0009), and human leukocyte antigen DR on CD14− CD16+ monocytes (OR = 1.0533, 95% CI: 1.0136–1.0945, *P* = .0081). Few significant heterogeneities or horizontal pleiotropies were observed. Through MR analysis, we identified distinct 5 types of immune cells that were positively correlated with the occurrence and development of FS, providing guidance for clinical intervention in FS.

## 1. Introduction

Frozen shoulder (FS), alternatively referred to as adhesive capsulitis, is a debilitating condition characterized by pain and gradual restriction of both active and passive movements of the shoulder joint.^[1]^ Rough estimates suggest that FS may affect approximately 2% to 5% of the general adult population,^[2]^ with a higher incidence in women aged 40 to 70.^[3]^ Other risk factors include diabetes,^[4]^ hyperthyroidism,^[5]^ infrequent immunization,^[6]^ and so on, which substantially increase the prevalence in these populations. Although there may be a natural tendency for symptom improvement in shoulder impingement, studies indicate that some patients may experience persistent symptoms and loss of motion.^[3]^ King et al^[7]^ identified the predicaments faced by patients with FS, including restricted mobility, sleep deprivation, delayed diagnosis, and uncertainty regarding effective treatment.

The etiology and pathogenesis of FS remain unclear and are commonly considered idiopathic. Current research primarily focuses on the role of the immune response. Patients with FS exhibit notable increases in the expression levels of IL-1α, IL-1β, COX-1, COX-2, and TNF-α within the joint capsule and subacromial bursa.^[8]^ In a prospective case-control study, Kabbabe et al^[9]^ found increased mRNA expression levels of inflammatory and fibroblast cytokines, such as IL-6 and IL-8, in the synovium of patients with FS. Histological analysis of affected tissues in patients with FS by Hand et al^[10]^ revealed evidence of infiltration by inflammatory cells, including T cells, B cells, macrophages, and mast cells. Several studies have indicated a close association between immune cells, inflammatory responses, and FS. However, further investigations are required to elucidate the precise pathological processes involved.

Mendelian randomization (MR) is a statistical technique that uses genetic variants as instrumental variables (IVs) to infer causal relationships between traits and diseases, particularly in situations in which randomized controlled trials are difficult to conduct.^[11]^ It leverages the principles of Mendel laws of inheritance, which state that alleles are randomly assigned as they are passed from parent to offspring. As a result, the genotype of the offspring is typically unrelated to confounding factors. Furthermore, genetic variation is fixed at the time of conception and generally predates the measured variables; therefore, the possibility of reverse causation is circumvented.^[12]^ The purpose of this study was to investigate the causality between immunophenotypes and FS, which may help gain a deeper understanding of the pathophysiological processes of FS and refine current preventative and therapeutic measures.

## 2. Methods

### 2.1. Data sources

**Figure 1.**
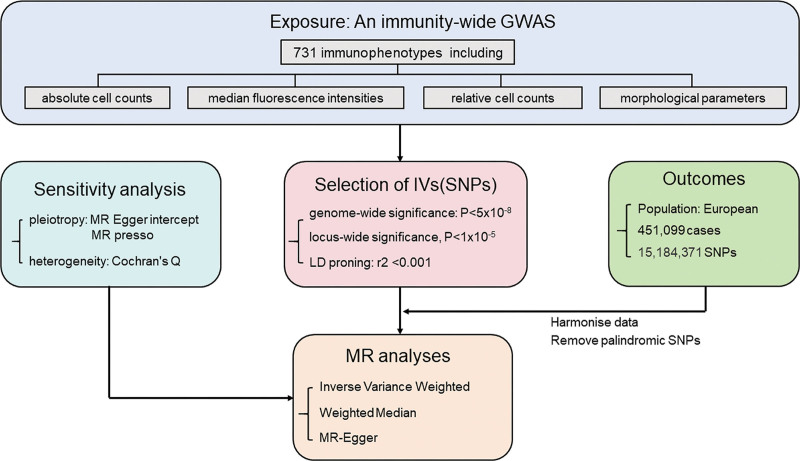
Mendelian randomization flowchart for immunophenotypes and frozen shoulder. IV = instrumental variable, MR = Mendelian randomization, SNP = single nucleotide polymorphisms.

We extracted GWAS summary statistics for 731 distinct immunological traits based on research published by Orrù et al.^[13]^ The 731 immunophenotypes examined included absolute cell counts, median fluorescence intensities, surface antigen levels, morphological parameters, and relative cell counts. We obtained summary statistics of FS from 451,099 individuals of European descent. In total, 15,184,371 single nucleotide polymorphisms (SNPs) were included in this study.^[14]^ Detailed information on the data sources is provided in Table 1. The goal of MR analysis is to uncover causal linkages between either single or multiple exposures and a specific outcome, achieved through the utilization of genetic variation as a proxy for risk factors. A comprehensive research methodology is outlined in Figure 1. The GWAS datasets employed are openly accessible to the public, with no individual-level data, and thus, do not require ethical approval.

**Table 1:** Details of the genome-wide association studies and datasets used in our analyses.

Exposure of outcome	Sample size	Ancestry	List of data download
Frozen shoulder	451,099	European ancestry	https://gwas.mrcieu.ac.uk/datasets/ebi-a-GCST90000512/
Genetic variation for immunophenotypes	3757	European ancestry	https://gwas.mrcieu.ac.uk/datasets/

### 2.2. Instrumental variables

**Figure 2.**
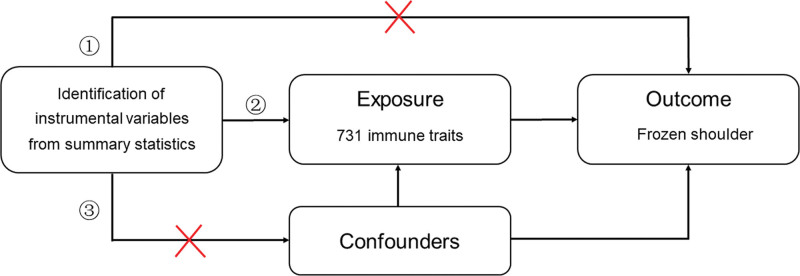
Assumptions in MR studies: a brief overview. MR = Mendelian randomization.

The selection of IVs in MR analysis requires satisfying 3 assumptions: IVs exhibit a direct relationship with exposure; IVs are not influenced by or correlated with confounding variables; and IVs exert an influence on the outcome solely through their association with exposure.^[15]^ The 3 major assumptions of MR are shown in Figure 2.

In this study, we applied a threshold of statistical significance set at *P* < 1.0 × 10^−5^ to ensure a strong association between IVs and immunophenotypes. We applied specific criteria (linkage disequilibrium^[16]^ *r*^2^ threshold <0.001 within 10,000 kb distance^[17]^) to ensure independence among IVs, aiming to minimize potential bias from genetic linkage disequilibrium. In genetic studies, the absence of a specific SNP in the outcome dataset can be addressed using proxy SNPs that are in linkage disequilibrium. To guarantee a robust association between the proxy SNPs and the target SNPs, we set an *r*^2^ threshold >0.8 to allow the proxy SNPs to effectively function as IVs in place of the target SNPs. It is crucial to highlight that palindromic SNPs, characterized by A/T or G/C alleles, may introduce ambiguity in identifying the causal allele in both exposure and outcome GWAS. To address this, we excluded palindromic SNPs with an effect allele frequency ranging from 0.3 to 0.7. To conduct the analysis, we utilized the “TwoSampleMR” package and leveraged 1000 Genomes EUR data. We calculated the strength of each IV with the *F*-statistic and discarded SNPs with *F* < 10 using the equation *F* = *β*^2^/SE^2^ to avoid bias from weak IVs.^[18]^ Moving forward, we focused on the prioritized SNPs with the most significant *P* value (*P* < .05) for each trait, resulting in the identification of 18,732 independent SNPs linked to 731 immune cell characteristics.

### 2.3. Statistical analysis

In our analysis, the inverse variance-weighted (IVW) method was primarily employed to investigate the causal effects between immune cells and FS, as it effectively mitigates outcome bias caused by heterogeneity. IVW, a weighted averaging method that computes the reciprocal of the variance for each estimate as weights, multiplies the weights by the corresponding estimate, and sums the weighted estimates to obtain the result, was successfully employed as a random-effects method to address any potential deviations arising from heterogeneity.^[19]^ The weighted median is a statistical method used in MR analysis that considers the weights of the data points to calculate the central value. The weighted median method enables an unbiased estimation of causal effects, even when only half of the IVs are effective.^[19]^ MR-Egger estimates causal effects by accounting for pleiotropy, providing insights into the causal linkages between an exposure factor and an outcome variable.^[19]^ When dealing with MR studies encompassing numerous genetic variants, sensitivity assessments such as the intercept of MR-Egger and MR-PRESSO analyses serve as complementary tools to strengthen the credibility of the findings and detect/correct for potential biases stemming from horizontal pleiotropy.^[19–21]^ Heterogeneity among IVs was assessed using Cochran *Q* test.^[22]^ The MR analysis was executed utilizing R (version 4.3.0) with the “TwoSampleMR” package (version 0.5.7).

## 3. Results

### 3.1. Selecting IV for MR analysis

After selecting and coordinating IVs, we utilized 28 SNPs for CD25++ CD45RA+ CD4 not regulatory T cell %CD4+ T cell, 22 SNPs for CD25++ CD45RA+ CD4 not regulatory T cell %T cell, 21 SNPs for CD127 on CD28+ CD4+ T cell, 21 SNPs for CD4 on human leukocyte antigen DR (HLA-DR)+ CD4+ T cell and16 SNPs for HLA-DR on CD14− CD16+ monocyte. The *F*-statistics for SNPs linked to 731 immune cell features spanned 19.55 to 2381.77, indicating no weak instrument bias. Detailed SNP information for each trait is provided in Tables 1 and 2, Supplemental Digital Content, http://links.lww.com/MD/N819.

### 3.2. The cause-and-effect relationship between FS development and immune phenotypes

**Figure 3.**
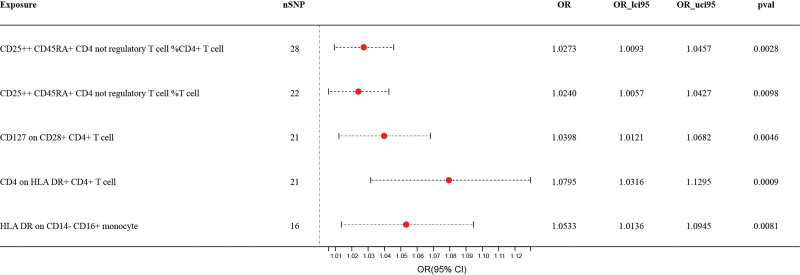
Forest plot: associations of genetically determined bacterial traits with frozen shoulder risk. CI = confidence interval, HLA-DR = human leukocyte antigen DR, OR = odds ratio.

Although most immune cells did not show a clear causal relationship with FS in this study, as indicated in Table 3, Supplemental Digital Content, http://links.lww.com/MD/N819, and Figure 3, we identified 5 immune cell traits that act as risk factors for FS, as shown in Table 4, Supplemental Digital Content, http://links.lww.com/MD/N819 (*P* < .01).

Through the IVW analysis method, we found a positive correlation between CD25++ CD45RA+ CD4 not regulatory T cell %CD4+ T cell (OR = 1.0273, 95% CI: 1.0093–1.0457, *P* = .0028) and FS, and the same was observed for CD25++ CD45RA+ CD4 not regulatory T cell %T cell (OR = 1.0240, 95% CI: 1.0057–1.0427, *P* = .0098). The MR-Egger analysis did not detect any significant outliers or indications of horizontal pleiotropy. The findings from the MR-Egger and weighted median analyses also support the conclusion drawn from the IVW. Likewise, the MR-PREESSO global test provided no evidence of horizontal pleiotropy (*P* = .3710 for CD25++ CD45RA+ CD4 not regulatory T cell %CD4+ T cells and *P* = .2830 for CD25++ CD45RA+ CD4 not regulatory T cell %T cell).

Similarly, the IVW results also indicated that both CD127 on CD28+ CD4+ T cells (OR = 1.0398, 95% CI: 1.0121–1.0682, *P* = .0046) and CD4 on HLA-DR+ CD4+ T cells (OR = 1.0795, 95% CI: 1.0316–1.2195, *P* = .0009) increased the risk of FS. The MR-Egger analysis did not identify any outliers or horizontal pleiotropy. In addition, MR-Egger and weighted median analyses further substantiated this positive association. The MR-PREESSO global test indicated the presence of horizontal pleiotropy (*P* = .5450 for CD127 on CD28+ CD4+ T cells and *P* = .3400 for CD4 on HLA-DR + CD4+ T cells).

The IVW method showed a positive correlation between HLA-DR and CD14− CD16+ monocyte (OR = 1.0533, 95% CI: 1.0136–1.0945, *P* = .0081), and FS risk was also observed. MR-Egger analysis showed no evidence of horizontal pleiotropy. However, in contrast to the aforementioned analysis of immune cells, while the results of the MR-Egger analysis supported IVW, the findings from the weighted median analysis were inconsistent with the IVW method and did not provide further support for a causal relationship between the two. Neither the intercept of the MR-Egger analysis (*P* = .2227) nor the MR-PREESSO global test (*P* = .3890) revealed horizontal pleiotropy.

The results of the IVW analysis consistently supported a positive correlation between these immune cells and FS risk. With the exception of the weighted median analysis for HLA-DR on CD14− CD16+ monocyte, which yielded results contrary to IVW, the MR-Egger and weighted median analyses for the remaining immune cells supported the conclusions drawn by IVW. This provides robust support for causal relationships between the studied variables. Furthermore, the lack of outliers and horizontal pleiotropy, as determined through the MR-Egger analysis and MR-PREESSO global test, confirmed the reliability of our findings (Tables 5 and 6, Supplemental Digital Content, http://links.lww.com/MD/N819). In order to provide additional evidence regarding the IVs of immunophenotypes, Cochran *Q* test was performed and found no significant heterogeneity among these variables, consistent with the previously discussed findings (Table 7, Supplemental Digital Content, http://links.lww.com/MD/N819).

## 4. Discussion

This 2-sample MR investigation uncovered a total of 5 immunophenotypes, including CD25++ CD45RA+ CD4 not regulatory T cell %CD4+ T cell, CD25++ CD45RA+ CD4 not regulatory T cell %T cell, CD127 on CD28+ CD4+ T cell, CD4 on HLA DR+ CD4+ T cell and HLA DR on CD14− CD16+ monocyte might be positively associated with the risk of FS.

Numerous studies have consistently demonstrated that chronic inflammation and tissue fibrosis are key mechanisms underlying the development of FS. Histologically, changes, such as increased vascularity, fibroblast proliferation, synovial thickening, and extracellular matrix deposition, have been identified to support this notion. Several inflammatory mediators, including TGF-β, IL-1, IL-6, IL-10, GM-CSF, M-CSF, PDGF, and TNF are involved in this process.^[23]^

Although some studies have suggested a potential association between abnormal activation of the immune system and FS pathogenesis, research specifically focusing on the role and mechanisms of T cells in FS is relatively limited. Most pertinent studies have been undertaken within the framework of other inflammatory states, such as arthritis, rather than specifically targeting FS. Immunocytochemical and histological staining of the FS biopsy material by Hand et al^[10]^ revealed the presence of inflammatory infiltration, including T-cell infiltration, with significant positive staining for CD45 (leukocyte common antigen). Liu et al^[24]^ also observed an increased percentage of CD45-immune cells in shoulder tissues, with the majority of the affected tissue consisting of lymphocytes, predominantly T cells. However, direct evidence supporting the relationship between the specific T cell subsets CD25++ CD45RA+ CD4 not regulatory T cell %CD4+ T cell and CD25++ CD45RA+ CD4 not regulatory T cell %T cell and FS has not been found.

The relationship between CD127 on CD28+ CD4+ T cells and FS has not yet been thoroughly investigated. CD127 serves as an integral component of the IL-7 receptor and plays a pivotal role in the development of T cells. Abnormal expression levels and dysfunctional behavior of IL-7 or its signaling pathway components are frequently linked to various chronic inflammatory autoimmune disorders, rheumatoid arthritis (RA) being one such prominent example. Aberrant expression and functional impairment of IL-7 or other components of the IL-7 signaling pathway are frequently linked to several chronic inflammatory autoimmune disorders, RA^[25]^ and type 1 diabetes^[26]^ being 2 such examples. The activation of CD28+ CD4+ T cells can promote the release of various inflammatory mediators, including IL-2, IL-4, IFN-γ, and TNF-α.

HLA-DR+ CD4+ T cells play crucial roles in inflammation and immune-related diseases such as colitis.^[27]^ Through further analysis of T cell composition based on single-cell RNA sequencing data, Akbar et al^[16]^ identified 8 T cell subgroups, including CD4+ T cells.

CD14− CD16+ monocytes are a nonclassical subset of monocytes that express HLA-DR more than classical and intermediate monocytes.^[28]^ Liu et al^[24]^ found that monocytes were one of the major infiltrating immune cells in FS. Research on the relationship between HLA-DR on CD14− CD16+ monocytes and sepsis has received more attention, but there is limited research specifically focusing on its association with FS. It has been observed that the population of CD14− CD16+ monocytes expands in the blood of patients with systemic infections, indicating their crucial role in rapid pathogen defense.^[29]^ In many clinical situations, diminished expression of HLA-DR on monocytes in the peripheral blood has been highly correlated with infection, and decreased HLA-DR expression levels have been identified as an indicator of immune suppression.^[30]^

Although the current understanding of the pathological mechanisms underlying FS is limited and controversial, involvement of the immune system cannot be overlooked. Multiple pieces of evidence support the presence and role of immune cells and inflammatory factors in the occurrence and progression of FS, consistent with our findings.

Our study has several limitations. First, the data source for the analysis was primarily based on the European population in the GWAS. While this helps mitigate potential biases arising from immune feature differences among different ethnicities to some extent, it is constrained in terms of its applicability to other populations. Further analysis that incorporates data from diverse ethnic backgrounds is necessary for extrapolation to other races. Second, the available GWAS data did not provide specific individual-level information such as height, weight, and age, which would have allowed for further stratification and layered MR analysis for FS. These additional factors could have contributed to more precise causal inferences and better control of the potential confounders. Third, the threshold used for selecting IVs in our screening process was set at *P* < 1.0 × 10^−5^, a relatively less stringent criterion compared to the conventional significance level of *P* < 5 × 10^–8^. This may have introduced potential false positives. Nonetheless, we calculated the *F*-statistic as a measure to evaluate the potential for bias in our IVs, and the assessment revealed negligible risk.

## 5. Conclusions

FS remains a disease with unclear mechanisms, and there is a lack of in-depth and systematic research in the relevant areas. The findings from our research could have significant clinical implications, contributing crucial insights toward the prevention, timely diagnosis, and management of FS. Although our study offers a profound understanding of the possible causal link between immune cells and FS via MR analysis, a deeper probe into the underlying biological mechanisms is imperative. Our findings may have important clinical implications and provide crucial insights into the prevention, early diagnosis, and intervention of FS.

## Author contributions

**Conceptualization:** Yinji Luo, Xinyu Wang, Bin Wang.

**Data curation:** Yinji Luo, Xinyu Wang.

**Formal analysis:** Yinji Luo, Xinyu Wang.

**Methodology:** Yinji Luo, Xinyu Wang.

**Software:** Yinji Luo, Xinyu Wang.

**Validation:** Yinji Luo, Xinyu Wang.

**Visualization:** Yinji Luo, Xinyu Wang.

**Writing—original draft:** Yinji Luo, Xinyu Wang, Bin Wang.

**Writing—review & editing:** Yinji Luo, Xinyu Wang, Bin Wang.

**Investigation:** Bin Wang.

**Project administration:** Bin Wang.

**Supervision:** Bin Wang.

## Supplementary Material



## References

[1] Neviaser AS Neviaser RJ. Adhesive capsulitis of the shoulder. J Am Acad Orthop Surg. 2011;19:536–42. 21885699 10.5435/00124635-201109000-00004

[2] Shah N Lewis M. Shoulder adhesive capsulitis: systematic review of randomised trials using multiple corticosteroid injections. Br J Gen Pract. 2007;57:662–7. 17688763 PMC2099674

[3] Date A Rahman L. Frozen shoulder: overview of clinical presentation and review of the current evidence base for management strategies. Future Sci OA. 2020;6:FSO647. 33312703 10.2144/fsoa-2020-0145 PMC7720362

[4] Huang YP Fann CY Chiu YH. Association of diabetes mellitus with the risk of developing adhesive capsulitis of the shoulder: a longitudinal population-based followup study. Arthritis Care Res (Hoboken). 2013;65:1197–202. 23281342 10.1002/acr.21938

[5] Wohlgethan JR. Frozen shoulder in hyperthyroidism. Arthritis Rheum. 1987;30:936–9. 3498494 10.1002/art.1780300815

[6] Redler LH Dennis ER. Treatment of adhesive capsulitis of the shoulder. J Am Acad Orthop Surg. 2019;27:e544–54. 30632986 10.5435/JAAOS-D-17-00606

[7] King WV Hebron C. Frozen shoulder: living with uncertainty and being in “no-man’s land”. Physiother Theory Pract. 2023;39:979–93. 35164645 10.1080/09593985.2022.2032512

[8] Lho YM Ha E Cho CH. Inflammatory cytokines are overexpressed in the subacromial bursa of frozen shoulder. J Shoulder Elbow Surg. 2013;22:666–72. 22999851 10.1016/j.jse.2012.06.014

[9] Kabbabe B Ramkumar S Richardson M. Cytogenetic analysis of the pathology of frozen shoulder. Int J Shoulder Surg. 2010;4:75–8. 21472067 10.4103/0973-6042.76966 PMC3063346

[10] Hand GC Athanasou NA Matthews T Carr AJ. The pathology of frozen shoulder. J Bone Joint Surg Br. 2007;89:928–32. 17673588 10.1302/0301-620X.89B7.19097

[11] Richmond RC Davey Smith G. Mendelian randomization: concepts and scope. Cold Spring Harb Perspect Med. 2022;12:a040501. 34426474 10.1101/cshperspect.a040501 PMC8725623

[12] Zheng J Baird D Borges MC. Recent developments in Mendelian randomization studies. Curr Epidemiol Rep. 2017;4:330–45. 29226067 10.1007/s40471-017-0128-6 PMC5711966

[13] Orrù V Steri M Sidore C. Complex genetic signatures in immune cells underlie autoimmunity and inform therapy. Nat Genet. 2020;52:1036–45. 32929287 10.1038/s41588-020-0684-4 PMC8517961

[14] Green HD Jones A Evans JP. A genome-wide association study identifies 5 loci associated with frozen shoulder and implicates diabetes as a causal risk factor. PLoS Genet. 2021;17:e1009577. 34111113 10.1371/journal.pgen.1009577 PMC8191964

[15] Skrivankova VW Richmond RC Woolf BAR. Strengthening the reporting of observational studies in epidemiology using Mendelian RANDOMIZATION: the STROBE-MR statement. JAMA. 2021;326:1614–21. 34698778 10.1001/jama.2021.18236

[16] Akbar M Crowe LAN McLean M. Translational targeting of inflammation and fibrosis in frozen shoulder: molecular dissection of the T cell/IL-17A axis. Proc Natl Acad Sci U S A. 2021;118:e2102715118. 34544860 10.1073/pnas.2102715118 PMC8488623

[17] Chen Y Shen J Wu Y. Tea consumption and risk of lower respiratory tract infections: a two-sample Mendelian randomization study. Eur J Nutr. 2023;62:385–93. 36042048 10.1007/s00394-022-02994-w PMC9427168

[18] Jiang X Zhou R He Y Zhu T Zhang W. Causal effect of serum 25-hydroxyvitamin D levels on low back pain: a two-sample Mendelian randomization study. Front Genet. 2022;13:1001265. 36212121 10.3389/fgene.2022.1001265 PMC9534573

[19] Hemani G Zheng J Elsworth B. The MR-Base platform supports systematic causal inference across the human phenome. Elife. 2018;7:e34408. 29846171 10.7554/eLife.34408 PMC5976434

[20] Bowden J Del Greco MF Minelli C. Assessing the suitability of summary data for two-sample Mendelian randomization analyses using MR-Egger regression: the role of the I2 statistic. Int J Epidemiol. 2016;45:1961–74. 27616674 10.1093/ije/dyw220 PMC5446088

[21] Bowden J Davey Smith G Haycock PC Burgess S. Consistent estimation in Mendelian randomization with some invalid instruments using a weighted median estimator. Genet Epidemiol. 2016;40:304–14. 27061298 10.1002/gepi.21965 PMC4849733

[22] Cohen JF Chalumeau M Cohen R Korevaar DA Khoshnood B Bossuyt PMM. Cochran’s Q test was useful to assess heterogeneity in likelihood ratios in studies of diagnostic accuracy. J Clin Epidemiol. 2015;68:299–306. 25441698 10.1016/j.jclinepi.2014.09.005

[23] Millar NL Meakins A Struyf F. Frozen shoulder. Nat Rev Dis Primers. 2022;8:59. 36075904 10.1038/s41572-022-00386-2

[24] Liu H Yu B Deng Z. Role of immune cell infiltration and small molecule drugs in adhesive capsulitis: novel exploration based on bioinformatics analyses. Front Immunol. 2023;14:1075395. 36875119 10.3389/fimmu.2023.1075395 PMC9976580

[25] van Roon JA Verweij MC Wijk MW. Increased intraarticular interleukin-7 in rheumatoid arthritis patients stimulates cell contact-dependent activation of CD4(+) T cells and macrophages. Arthritis Rheum. 2005;52:1700–10. 15934068 10.1002/art.21045

[26] Harrison C. Autoimmune disease: targeting IL-7 reverses type 1 diabetes. Nat Rev Drug Discov. 2012;11:599. 22850777 10.1038/nrd3805

[27] He JY Kim YJ Mennillo E. Dysregulation of CD4(+) and CD8(+) resident memory T, myeloid, and stromal cells in steroid-experienced, checkpoint inhibitor colitis. J ImmunoTher Cancer. 2024;12:e008628. 38642938 10.1136/jitc-2023-008628 PMC11033653

[28] Kapellos TS Bonaguro L Gemund I. Human monocyte subsets and phenotypes in major chronic inflammatory diseases. Front Immunol. 2019;10:2035. 31543877 10.3389/fimmu.2019.02035 PMC6728754

[29] Chomel L Vogt M Demiselle J. TLRs1-10 protein expression in circulating human white blood cells during bacterial and COVID-19 infections. J Innate Immun. 2024;16:216–25. 38461810 10.1159/000536593 PMC11001289

[30] Padovani CM Yin K. Immunosuppression in sepsis: biomarkers and specialized pro-resolving mediators. Biomedicines. 2024;12:175. 38255280 10.3390/biomedicines12010175 PMC10813323

